# Dynamics of Executive Functions, Basic Psychological Needs, Impulsivity, and Depressive Symptoms in American Football Players

**DOI:** 10.3389/fpsyg.2019.02409

**Published:** 2019-10-31

**Authors:** Yahel E. Rincón-Campos, Javier Sanchez-Lopez, Jeanette M. López-Walle, Xóchitl Ortiz-Jiménez

**Affiliations:** ^1^Facultad de Organización Deportiva, Universidad Autónoma de Nuevo León, Monterrey, Mexico; ^2^Department of Neuroscience, Biomedicine and Movement Sciences, University of Verona, Verona, Italy; ^3^Facultad de Psicología, Universidad Autónoma de Nuevo León, Monterrey, Mexico

**Keywords:** inhibition, frustration, football, impulsivity, motivational processes

## Abstract

Executive functions play an important role in sports since the ability to plan, organize, and regulate behavior to reach an objective or goal depends on these functions. Some of the components of executive functions, such as inhibition of impulsive behavior and cognitive flexibility, are necessary for contact sports (e.g., American football) to carry out successful plays on the sports field. Executive functions have been studied in the sporting environment, but their relationship with the athletes’ basic psychological needs (BPN), such as autonomy, competence, and relatedness, remains unexplored. Due to the importance of motivational processes over cognitive functions and in the generated adaptive results in athletes, this relationship should be taken into account. Therefore, the aim of this study was to analyze and compare executive functioning and psychological need thwarting overimpulsivity and psychological distress, before and after the season (4 months) in 28 undergraduate football players. Neuropsychological and psychological tests were applied. The results showed that there was an improvement in inhibition and planning at the end of the season. There was also an increase in attention and motor impulsiveness, and a decrease in need thwarting at the end of the season. A positive association between executive function, impulsiveness, psychological needs, and affective symptoms were also found. Our findings reveal the dynamics of sport-related psychological variables throughout the sport season in American football players, the association of these for the achievement of sport success, and the importance of encouraging proper management of emotions.

## Introduction

The development of maximum sporting capacities depends on different components, such as physiological, technical, cognitive, emotional, personality-related, and motivational factors. The harmonic interaction of these components results in optimal trainability (Harre, 1987) and in the attainment of sport mastery.

The cognitive component refers to the set of mental processes carried out by individuals. Among these processes, executive functions are the mental skills essential to perform effective, creative, and socially accepted behavior (Lezak, 1995). Accordingly, executive functions are considered abilities that make it possible to organize and plan a task, appropriately select objectives, start and keep in mind an action plan, be flexible in strategies to reach a goal, or inhibit irrelevant stimuli (Shallice, 1994; Soprano, 2003; Banich, 2009). Also, executive function is a construct that comprises a series of cognitive abilities involved in the control of thought and behavior (Zelazo and Carlson, 2012). This is why, in the sporting environment, these abilities or functions are involved in a variety of tasks performed within physical practice (Lezak, 1993; Hillman et al., 2003; Davidson et al., 2006). The benefits derived from physical activity comprise not only physiological or physical health improvement but also better cognitive function, which has been a topic of great interest. Several studies have reported a positive association between cognitive function and physical activity, e.g., improvement in planning ability (Davis et al., 2011), better inhibitory control (Hillman et al., 2009; Wu et al., 2011), improvement in working memory (Kubesch et al., 2009; Rigoli et al., 2012), greater cognitive flexibility (Buck et al., 2008), and better responses in cognitive tests measuring aspects related to executive function (Stroth et al., 2009). The impact of physical practice on cognitive performance, and specifically on executive functions, may be derived from cognitive demands inherent to exercise, physiological changes produced in the brain, and the existing cognitive implications when performing a complex motor task (Castelli et al., 2007; Tomporowski et al., 2008; Best, 2010). Collective sports entail tasks with a great cognitive load, such as collaborating with teammates, anticipating the actions of opponents, developing strategies to achieve a successful play, or inhibiting secondary stimuli, and focusing on important ones, which, in turn, stimulate executive functions since great cognitive involvement is required that generates greater brain activation.

Another important component for sports performance, closely linked to executive functioning, is impulsiveness, which plays an important role in personality theories (Eysenck and Eysenck, 1985; Cloninger et al., 1991). Impulsiveness is defined as a predisposition to quickly and spontaneously react to both internal and external stimuli without considering the negative consequences for oneself and others. In clinical settings, this variable is associated with several psychiatric issues (Eysenck and Eysenck, 1978; Barratt, 1983, 1985, 2004; Moeller et al., 2001; Chahín, 2011; American Psychiatric Association [APA], 2013). However, in the sporting environment, impulsiveness has had different connotations, both positive (functional) and negative (dysfunctional), and has been associated with high levels of aggressiveness, functionality, and athlete success (Hickmann, 2004), as well as predisposition to a specific sport choice (Svebak and Kerr, 1989).

From a motivational perspective, the self-determination theory (Ryan and Deci, 2017) and, more specifically, the subtheory of basic psychological needs (BPN) sustain that needs play an important role in the development of psychological well-being and optimal functioning (Ryan and Deci, 2000) in athletes’ performance. The theory postulates the existence of three BPN: autonomy, competence, and relatedness. This theory has a “dark side” in which frustration of BPN is analyzed. Frustration is referred to, in Bartholomew et al. (2011b), as an experience, a “state of mood.” Autonomy satisfaction refers to the experience of self-determination, full willingness, and volition when carrying out an activity. In contrast, autonomy frustration involves feeling controlled through externally enforced or self-imposed pressures (deCharms, 1968; Deci and Ryan, 1985). Relatedness satisfaction refers to the experience of intimacy and genuine connection with others (Ryan, 1995), while relatedness frustration involves the experience of relational exclusion and loneliness (Bartholomew et al., 2011a). Competence satisfaction involves feeling effective and capable to achieve desired outcomes (Deci, 1975; Ryan, 1995), while competence frustration involves feelings of failure and doubt about one’s efficacy (Bartholomew et al., 2011a); when thwarted, these needs are associated with dysfunction and are indicators of discomfort/ill-being (Stebbings et al., 2012; Vansteenkiste and Ryan, 2013). Concretely in sports, they are associated with symptoms of physical and psychological discomfort (Mars et al., 2017). Moreno-Murcia and Cervelló (2010) state that the lack of satisfaction of these basic needs leads to a series of cognitive, affective, and behavioral consequences, as well as exhaustion (Bartholomew et al., 2011b), somatic complaints (Bartholomew et al., 2014), burnout (Balaguer et al., 2012; Castillo et al., 2012; Bartholomew et al., 2014), negative affect (Gunnell et al., 2013), and affective disorders, such as anxiety and depression (Jowett and Felton, 2014).

From a physiological perspective, sport training involves variations throughout the training period for competition purposes, and from the pre-competitive to the competitive season (see, for example, Koutedakis, 1995); these variations are specific to every sports discipline and depend on its demands and the organization of training sessions. Intuitively, these physiological variations are expected to occur along with the described sport-related psychological components. To our knowledge, there are no studies investigating these variations. On the other hand, as stated at the beginning of this section, the harmonic interaction of the different components that make up sport performance guarantees optimal trainability. In general, this association between executive function and impulsiveness has been previously demonstrated (Hickmann, 2004). Specifically, in the sporting environment, the association between thwarting of psychological needs and indicators of psychological distress, such as depression and anxiety, was also studied (Gunnell et al., 2013; Jowett and Felton, 2014). However, the existing evaluations of the interaction between these components are not systematic, in particular among cognitive and motivational components, although some hypotheses linking executive functioning and satisfaction of psychological needs, particularly autonomy, have been proposed (Ryan and Deci, 2006).

Therefore, the first objective of this study was to compare the performance of executive function, impulsiveness, psychological needs thwarted, and indicators of psychological distress, before and after the season in a semi-professional, college football team. The second objective of this study was to provide evidence on the association of these sport-related psychological components in the population described. If we consider the dynamics of a sports environment during the season, then we can suppose that all the components implicated in sports performance such as cognition, motivation, and emotion, should be dynamic as well; i.e., we could hypothesize differences in these components between the beginning and end of the sports season. On the other hand, if a harmonic interaction of these components is necessary for optimal sport performance, as we stated above, then we could hypothesize a significant association between these components, mainly in their relationship with cognitive functioning, which has been less explored in previous studies.

## Materials and Methods

### Participants

Twenty-eight college football players from a semiprofessional team with mean age = 21.77 (*SD* = 1.77), mean years of schooling = 14.39 (*SD* = 1.87), and mean of months of sports practice = 153.14 (*SD* = 63.25) were recruited. The participants included in this study did not report a history of psychiatric or neurological pathology. All the athletes participated voluntarily, and after being fully informed about the experimental procedures and their rights, individual informed consent to participate in the study was obtained. Participants were rewarded with a detailed report of the results of their psychological and neuropsychological tests. The study was carried out in accordance with the Helsinki declaration and was presented to the American Football Team Commission who gave their approval.

### Instruments

To measure Executive Functions, the following tests were applied:

(a) The Montreal Cognitive Assessment (MoCA, version 7.1) validated for Latin America (Loureiro et al., 2018). MoCA evaluates executive functions, visuospatial ability, memory, attention, concentration and working memory, language, and orientation. The maximum score is 30 points, and the cutoff score for subjects without cognitive impairment is 26. The administration time is approximately 10 min.

(b) The Trail Making Test (TMT) included in the Halstead–Reitan Battery (Reitan and Wolfson, 1985) measures visual search speed, scanning, attention, sequencing, and cognitive flexibility; it consists of two parts: TMT-A and TMT-B. In TMT-A, the participant is instructed to draw a line to connect 25 numbers in ascending order (1, 2, 3 … 25), which are randomly distributed throughout a sheet of paper (Letter size). In TMT-B, the participant alternates numbers and letters in an ascending sequence (1-A, 2-B, 3-C … 12-L, 13). The total time to perform each test, in seconds, is recorded. The time limit for TMT-A is 100 s, and 300 s for TMT-B. For this study, the number of correct answers per test (numbers/letters correctly connected in ascending order) was also recorded, and error types such as omissions (number or letter), order (number or letter), errors of perseverance, and error corrections (going back) were qualitatively analyzed.

(c) The Stroop Effect subtest of the Neuropsychological Battery of Executive Functions and Frontal Lobes Version 2 (BANFE-2; Flores et al., 2012) was used. This subtest evaluates the ability of the participant to inhibit an automatic response and select a response based on arbitrary criteria (word color or reading the word). The maximum score is 84 with an administration time of up to 5 min. Correct answers (number of words correctly read) and the time used to complete the test (in seconds) were recorded.

To measure impulsiveness, the Barratt Impulsiveness Scale Version 11 for adults (BIS-11; Patton et al., 1995) was applied. The BIS-11 is a Likert-type scale composed of 30 items with four response options regarding frequency: 1 = *rarely or never*, 2 = *occasionally*, 3 = *often*, 4 = *always or almost always*. The total score ranges from 30 to 120 and evaluates global impulsiveness, as well as three second-order factors: attentional impulsiveness (5, 6, −9, 11, −20, 26, 28), motor impulsiveness (2, 3, 4, 16, 17, 19, 21, 22, 23, 25, −30) and unplanned impulsiveness (−1, −7, −8, −10, 12, −13, 14, −15, 18, 27, −29). The Spanish version validated in Chilean population was applied (Cronbach’s alpha of 0.77; Salvo and Castro, 2013).

To measure psychological needs, the Psychological Need Thwarting Scale (PNTS; Bartholomew et al., 2011b) in its version validated for the Mexican context was applied (Cronbach’s alpha of 0.95; López-Walle et al., 2013). PNTS is a Likert-type scale composed of 12 items, which can be classified into a global dimension of need thwarting and three factors: autonomy (items 1, 3, 5, and 7), relatedness (items 4, 8, 10, and 12), and competence (items 2, 6, 9, and 11). The response options range from 1 to 7 in ascending order (from 1 = *totally disagree* to 7 = *totally agree*), and the maximum scores range from 7 to 84 in the global dimension, and from 4 to 28 in each factor.

To measure the affective state, two scales were used:

(a) The Spanish version (Sanz et al., 2003) of the Beck Depression Inventory-II (BDI-II; Beck et al., 1996) validated in non-clinical samples of the Mexican general population and undergraduate students. This validation showed high internal consistency in the general factor (Cronbach’s alpha = 0.9). BDI-II is a self-reported inventory that evaluates the intensity of depressive symptomatology through 21 items with four response options (from 0 to 3) ordered from low to high intensity of symptoms. Scores range from 0 to 63 points (0–13 = *without depression*, 14–19 = *mild depression*, 20–28 = *moderate depression*, and 29–63 = *severe depression*). The inventory is answered considering the mood during the two previous weeks including the administration day.

(b) The Generalized Anxiety Disorder questionnaire 7 (GAD-7) has been validated in the Spanish population with a Cronbach’s alpha of 0.93 and a high criterion and construct validity (García-Campayo et al., 2009). GAD-7 screens for generalized anxiety disorder testing the presence of symptoms listed in the DSM-V. GAD-7 is a Likert-type scale of seven items with four response options: 0 = *never*, 1 = *several days*, 2 = *more than half the days*, and 3 = *nearly every day*. The scores range between 0 and 21 and are classified into four categories depending on the level of anxiety: 0–4 = *minimum*, 5–9 = *mild*, 10–14 = *moderate*, and greater than 14 = *severe*.

### Procedure

Semi-professional college football players were invited to participate in the research project, which consisted of two evaluations during the season, i.e., Time 1 = pre-season, and Time 2 = post-season. In the first evaluation (Time 1), the procedure was explained and informed consent per participant was obtained, after which a general data questionnaire was administered (demographics, current sport, and clinical history); finally, the cognitive, affective, impulsiveness, and psychological needs assessments described above were administered. This was done in classrooms of their university with the necessary facilities (a desk, two comfortable chairs, good lighting, and air conditioning) at specific times (morning/evening) for two continuous weeks. The second evaluation (Time 2) was carried out at the end of the season (5 months after the first session), and consisted of the application of the same protocol, except for the presentation, informed consent, and interview.

### Data Analysis

Statistical analyses were performed using SPSS software version 24 (Ibm Corporation, 2016). To analyze the normal distribution of our data, the Kolmogorov–Smirnov test for one sample was applied to every variable for pre- and post-season evaluations.

The variables that did not reach the normal distribution criterion were subjected to mean-comparison analyses for related samples using the Wilcoxon signed-rank test: cognitive evaluation (Total MoCA, Stroop correct answers, Stroop time, TMT-A time, TMT-A correct answers, TMT-B time, and TMT-B correct answers), depression (Total BDI-II), anxiety (Total GAD-7), and psychological need thwarting (Autonomy, Relatedness, and Competence). FDR (False Discovery Rate) corrections for multiple comparisons were applied to the variables belonging to the same construct: mental flexibility (TMT-A and TMT-B) and psychological need thwarting (Autonomy, PNT-A; Relatedness, PNT-R; and Competence, PNT-C).

For variables that reached normality, *t-*tests for related samples were used: psychological need thwarting (total PNT). For impulsiveness (total BIS-11, attentional impulsiveness, motor impulsiveness, and unplanned impulsiveness), a two-way ANOVA test was performed with time (pre-season and post-season) and impulsiveness (attention, motor and non-planned) as factors. The Greenhouse–Geisser correction for sphericity violation was applied when there was more than one degree of freedom in the numerator.

Finally, to test the prediction and association between variables, a series of stepwise multiple linear regressions, to evaluate the explanatory function of the independent variable on dependent variables, were performed according to the model explained below. In addition, Pearson correlation coefficients between the variables included in the model were obtained.

(a) To test whether *executive functions* predicted *impulsiveness*, the independent variables used were total MoCA, TMT-A/B (correct answers and time) and Stroop (correct answers and time); total and partial scores of *impulsiveness* (attention, motor and non-planned) were used as dependent variables.
(b) To test whether *executive functions* predicted *autonomy* (dependent variable), the independent variables used were Total MoCA, TMT A/B (correct answers and time), and Stroop (correct answers and time).
(c) To test whether *impulsiveness* (dimensions) predicted *autonomy* (dependent variable), the independent variables used were attentional impulsiveness, motor impulsiveness, and unplanned impulsiveness.
(d) To test whether *psychological need thwarting* (factors) predicted *depression* (dependent variable), the independent variables used were autonomy, relatedness, and competence.
(e) To test whether *psychological needs thwarting* (factors) predicted *anxiety* (dependent variable), the independent variables used were autonomy, relatedness, and competence.

## Results

### Normality Test

Most of the variables did not show a normal distribution (see Table 1); therefore, different statistical tests (parametric and non-parametric) were used for comparisons in further analyses.

**TABLE 1:** Normality test results.

	**Pre-season**		**Post-season**	
		
**Variables**	**K-S**	***p*-value**	**K-S**	***p*-value**
**Executive functions**				
MoCA total	0.19	0.009	0.17	0.023
Stroop test hits	0.21	0.002	0.28	<0.001
Stroop test time	0.17	0.027	0.10	0.200
TMT-A time	0.25	<0.001	0.23	0.000
TMT-B time	0.20	0.003	0.25	0.000
**Psychological need thwarting**				
Autonomy	0.14	0.150	0.17	0.028
Relatedness	0.19	0.008	0.20	0.004
Competence	0.17	0.028	0.18	0.014
PNT total	0.10	0.200	0.16	0.062
**Affective assessment**				
GAD-7	0.17	0.029	0.14	0.129
BDI-II	0.21	0.002	0.18	0.015
**Impulsivity**				
BIS-11 total	0.11	0.200	0.13	0.184
Attention impulsiveness	0.11	0.200	0.10	0.200
Motor impulsiveness	0.12	0.200	0.11	0.200
Non-planning impulsiveness	0.10	0.200	0.10	0.200

*Results of the test to evaluate normal distribution of the variables using Kolmogorov–Smirnov are shown for the pre- and post-season evaluation; *n* = 28. MoCA = Montreal Cognitive Assessment; TMT = Trail Making Test; PNT = Psychological Need Thwarting; GAD-7 = Generalized Anxiety Disorder-7 Scale; BDI-II = Beck Depression Inventory-II; BIS-11 = Barratt Impulsiveness Scale-11.*

### Pre–Post Comparisons

The purpose of these analyses was to evaluate the differences between pre- and post-season in the scores of cognition, impulsiveness, psychological needs, and affective symptoms in this group of athletes. For the analysis of variables with a non-normal distribution, results showed a significant difference in the percentage of correct answers in the Stroop test, where athletes showed higher scores in post- compared to pre-season, as well as a greater number of correct answers in the TMT-B in the post-season and longer time spent executing TMT-A and TMT-B in post-season. Total Psychological Need Thwarting and Relatedness decreased significantly in post-season (see Table 2).

**TABLE 2:** Comparison of pre- and post-season.

	**Pre-season**	**Post-season**			
					
**Variable**	**Median (IQR)**	**Median (IQR)**	***Z***	***p*-value**	**Effect Size (*r*)*^*x*^***
**Executive functions**					
MoCA total	27.00 (2.00)	26.00 (2.75)	–1.69	0.090	–
Test of stroop hits	98.80 (3.27)	99.40 (2.08)	–2.17	^*^0.029	0.41
Test of stroop Time	64.50 (14.50)	66.00 (14.75)	–0.02	0.980	–
TMT-A	24.00 (10.00)	31.00 (8.00)	–3.42	^*^0.002^a^	0.65
TMT-B	48.00 (34.00)	65.00 (26.00)	–2.41	^*^0.016^a^	0.45
**Psychological needs thwarting**					
Autonomy	9.50 (3.75)	8.50 (8.75)	–0.67	0.750^a^	–
Relatedness	9.50 (8.50)	7.50 (5.00)	–2.67	^*^0.021^*^	0.50
Competence	8.50 (5.00)	8.00 (9.00)	–0.01	0.987^a^	–
**Affective assessment**					
GAD-7	3.00 (5.00)	3.00 (4.75)	–0.85	0.391	–
BDI-II	5.00 (8.00)	6.50 (7.75)	–0.37	0.705	–

	**Mean (SD)**	**Mean (SD)**	***t*(df)**	***p-*value**	**Effect Size (Cohen’s *d*)^*x*^**

PNT total	28.64 (9.35)	26.28 (12.69)	*t*(27) = 1.331	0.194	–

*IQR = interquartile range. ^*a*^FDR correction was used to correct for multiple comparison in variables belonging to the same construct. ^*x*^Effect size (*r*) is only reported when significant results were found. ^∗^significant results with *p* < 0.05.*

Parametric analysis of normally distributed variables showed significant differences in main effects of Impulsiveness and Time and in Impulsiveness × Time interaction. Pairwise comparisons revealed that Motor Impulsiveness was greater than Unplanned Impulsiveness and Attention Impulsiveness. The Total Impulsiveness score increased in post- compared to pre-season. The *post hoc* analysis of Time × Impulsiveness interaction showed that, in pre-season, Motor Impulsiveness was greater than Attention Impulsiveness and Unplanned Impulsiveness; in the post-season evaluation, Motor Impulsiveness was greater than Unplanned Impulsiveness. Finally, Motor Impulsiveness and Attentional Impulsiveness increased in post-season (see Table 3).

**TABLE 3:** Two-way ANOVA (pre/post-season × impulsivity dimensions) results.

**Main Effect**	***F***	***Df***	***p***	**$\eta_{p}^{2}$**	**ε**	**Power estimate**
Impulsivity	6.98	1.7, 48.9	0.003	0.206	0.89	0.88

**Pairwise comparisons**						

	Motor > attention		MD = 0.17		*p* = 0.009	
	Motor > non-planning		MD = 0.17		*p* = 0.002	
Time	17.97	1.00, 27.0	<0.001	0.400	1.00	0.98

**Pairwise comparisons**						

Time 2 > Time 1			MD = 0.14		*p* = 0.000	

**Interaction**	***F***	***Df***	***p***	**$\eta_{p}^{2}$**	**ε**	**Power estimate**

Impulsivity × time	4.58	1.95, 52.8	0.015	0.145	0.978	0.74

**Pairwise comparisons**						

	Motor time 1 > attention time 1		MD = 0.24		*p* ≤ 0.001	
	Motor time 1 > non-planning Time 1		MD = 0.19		*p* = 0.024	
	Motor time 2 > non-planning Time 2		MD = 0.19		*p* = 0.010	
	Attention time 2 > attention time 1		MD = 0.26		*p* ≤ 0.001	
	Motor time 2 > motor time 1		MD = 0.11		*p* = 0.023	

### Prediction and Association Between Variables

#### Pre-season

Pre-season linear regression analyses for the first step (a) showed that among the Executive Function variables, time of Stroop test significantly predicted Unplanned Impulsiveness; likewise, correct answers of TMT-A predicted Total and Motor Impulsiveness. In the fourth step (d), Psychological Need Thwarting of Autonomy predicted Depression. Finally, for the fifth step (e), the Autonomy factor of Psychological Need Thwarting predicted Anxiety (see Table 4).

**TABLE 4:** Pre-season stepwise linear regressions.

**Independent**	**Dependent**	***R*^2^**	***F* (df1, df2)**	***P***	**β (*p*-value)**
Stroop time	Non-planning	0.152	4.670 (1, 26)	0.040	−0.39 (0.040)
Autonomy	Depression	0.243	8.363 (1, 26)	0.008	0.49 (0.008)
Autonomy	Anxiety	0.178	5.636 (1, 26)	0.025	0.42 (0.025)

In the second step (b), in addition to the predictability using Unplanned Impulsiveness, a significant correlation between Stroop test time and Total Impulsiveness was found. For the third step (c), no predictions were found but Total Impulsiveness and Unplanned Impulsiveness correlated with Autonomy (PNT-A). In the fourth step (d), the Competence factor of Psychological Need Thwarting correlated with Depression (see Table 5).

**TABLE 5:** Pre-season bivariate correlations.

**Bivariate correlations**	***r* (Pearson)**	***p-*value**
(a) Executive functions – impulsivity		
Stroop time – BIS-11	−0.362	0.029
Stroop time – non-planning	−0.390	0.020
(c) Impulsivity – autonomy		
BIS-11 – autonomy	0.341	0.038
Non-planning – autonomy	0.363	0.029
(d) PNT – depression		
Autonomy – BDI-II	0.493	0.004
Competence – BDI-II	0.356	0.032
(e) PNT – anxiety		
Autonomy – GAD-7	0.422	0.013

The second step (b) for pre-season showed no predictions or correlations between cognitive factors and Autonomy (PNT-A).

#### Post-season

Linear regression analyses for post-season showed results similar to those found in the pre-season analysis. Specifically, for the first step (a), TMT-B time predicted Total and Unplanned Impulsiveness, and in the fourth step (d), the Autonomy factor of Psychological Need Thwarting predicted Depression (see Table 6).

**TABLE 6:** Post-season stepwise linear regressions.

**Independent**	**Dependent**	***R*^2^**	***F* (df1, df2)**	***p***	**β (*p*-value)**
TMT-B	BIS-11 total	0.168	5.049 (1, 25)	0.034	0.41 (0.034)
TMT-B	Non-planning	0.358	13.940 (1, 25)	0.001	0.59 (0.001)
Autonomy	Depression	0.209	6.862 (1, 26)	0.015	0.45 (0.015)

For the third step (c), no predictions were found but Motor Impulsiveness correlated with Autonomy. In the fourth step (d), the Competence factor of Psychological Need Thwarting correlated with Depression, and in the fifth step (e), a correlation between the Autonomy factor and Anxiety was found (see Table 7).

**TABLE 7:** Post-season bivariate correlations.

**Bivariate correlations**	***r* (Pearson)**	***p*-value**
(a) Executive functions – impulsivity		
TMT-B – BIS-11	0.410	0.017
TMT-B – non-planning	0.598	0.000
(c) Impulsivity – autonomy		
Motor impulsiveness – autonomy	0.328	0.044
(d) PNT – depression		
Autonomy – BDI-II	0.457	0.007
Competence – BDI-II	0.356	0.032
(e) PNT – anxiety		
Autonomy – GAD-7	0.370	0.026

For the second step (b) of post-season, neither predictions nor correlations between cognitive factors and Autonomy (PNT-A) were found.

## Discussion

The present study had two objectives: on one hand, to compare and analyze the behavior of executive functioning, impulsiveness, psychological need thwarting, and indicators of psychological distress (depression and anxiety) before and after the season in a semi-professional college American football team as part of the sport-related psychological variations that occur throughout a training period, and, on the other hand, to provide evidence on how these components are associated to integrate sport performance.

In general, the results showed, as hypothesized, that the components evaluated varied throughout the sports season, as observed for executive functions, which improved in the post-season, with a significantly increased number of correct answers in the tests that measured inhibition (Stroop) and planning (TMT-A/B). It is well known that an increase in the level of physical activity is associated with improvements in physical health and cognitive functioning in populations either with or without psychological difficulties (Colcombe et al., 2004; Booth et al., 2013). In the last decades, numerous studies have examined the association between physical activity and cognitive functioning. Recent results indicated that physical activity not only improves general cognitive function but also improves performance in tasks that rely on executive functioning (Khan and Hillman, 2014; Tomporowski et al., 2015; Donnelly et al., 2016). The different dimensions of cognitive performance, such as processing speed, planning, and control strategies, and working memory, may also improve with physical exercise and regular physical activity (Romero et al., 2017). Likewise, this improvement in executive functioning in athletes who are considered experts, that is, with more experience in the sport that they practice, as occurs in the sample of the present study, can be understood and explained from the perspective of increased neuronal flexibility. These expert athletes carry out sensory, perceptual, and cognitive processes in a more efficient way, which may be reflected in their performance on the sports field. Spinelli et al. (2011) established the concept of neural flexibility to explain this phenomenon of sports excellence in expert athletes; e.g., a study in martial arts athletes found that expert athletes showed better performance in tasks that evaluated executive functions compared to novice and non-athletes (Sanchez-Lopez et al., 2014, 2016). Though the mentioned study compared two groups cross-sectionally, its consideration, in order to explain the present results, may suggest that physical and technical–tactical training throughout the season favors improvements in cognitive functioning of athletes, perhaps as a process of greater general cognitive flexibility resultant from sport training or as part of a mechanism of greater and better adaptability to the demands of championship-driven sports tasks.

Increases in impulsiveness were found in the post-season evaluation, with motor and attentional impulsiveness showing higher scores. In a study conducted by Svebak and Kerr (1989), where impulsiveness played a role in the preference of sports type such as “explosive,” “endurance,” “paratelic,” and “non-paratelic,” the authors concluded that athletes involved in explosive and telic sports (search for excitement) types, where American football belongs, showed higher levels of total and unplanned impulsiveness (BIS-11). Although the results of Svebak and Kerr are in agreement with the high levels of impulsiveness found in American football athletes in the present study, their findings do not explain the variations of these scores in the post- compared to the pre-season. To our knowledge, there are no studies that analyze the variations of impulsiveness over time in an athlete population. We propose that the variations in impulsiveness throughout the season may result from increases in so-called functional impulsiveness, as reported in the study conducted by Hickmann in 2004 with NFL players where most players scored higher on functional, compared to dysfunctional, impulsiveness in the Functional/Dysfunctional Impulsiveness Inventory (FDI). In turn, these scores positively correlated with higher scores in BIS-11. In this same study, Hickmann found that players described as more impulsive also showed higher levels of sports success. From this point of view, in the present study, the athletes likely show a sort of increase in functional impulsiveness that enhances the availability of physical and psychological resources to efficiently solve the sports task.

Psychological need thwarting decreased significantly in the post-season; that is, lower thwarting perception was found in the second evaluation compared to the first. On one hand, some longitudinal studies that assessed BPN have found that the three psychological needs remained stable at moderate to high levels during competitive seasons (Adie et al., 2012). However, another study found that over a competition period, players’ feelings of vitality increased as a result of overcoming the continuous challenges of competition; in other words, thwarting perception decreased. In this study, the decrease in thwarting of BPN may be attributed to the fact that it is an active process, in which the training style contributed throughout the season, and as the games were played, the feeling of affection toward and from the team increases, especially when winning. It is important to consider that the American football players recruited for this study have relentlessly been league champions for several years. This may increase their sense of belonging and group cohesion, which has a positive effect not only on the group but also on the individuals.

Regarding the association between the components evaluated, we found that executive functions predict impulsiveness in both evaluation times (pre- and post-season) with a different direction in the linear regression corresponding to pre-season, in which athletes showed greater planning performance (TMT-A correct answers) and inhibitory control (Stroop time), thus predicting better planning ability (less unplanned impulsiveness) and less impulsiveness (Total BIS-11); i.e., longer times spent by players in the Stroop test corresponded with better planning ability. On the other hand, in the post-season evaluation, results showed that if an athlete has better processing speed and cognitive flexibility (TMT-B) and greater cognitive control for response planning, BIS-11 Total/no planning may be predicted. Similarly, another study found that soccer players evaluated with the Barratt scale and the Tower of London obtained mean scores that, in turn, showed that longer planning time was associated with better problem solving (Culbertson and Zillmer, 2001).

On the other hand, executive functions failed to predict or correlate with autonomy thwarting. A publication by Ryan and Deci in 2006 describes the neurobiological basis of autonomy by explaining that autonomy requires the coordination among prefrontal cortical regions responsible for regulation, striatal–thalamic regions to promote or inhibit motivation, and inputs from the hippocampus and amygdala to provide contextual and affective information (e.g., Bradley, 2000; Chambers et al., 2003 cited by Ryan and Deci, 2006). Walton et al. (2004) stated that neuronal mechanisms operate depending on whether the medium informs us what to do or whether we act autonomously. Therefore, Walton et al. (2004) and Ryan and Deci (2006) suggest that executive functions must be both selective and fully informed by affective and memory-related processes to support and improve autonomy, which may be a reason why no correlation between these variables was found. Nevertheless, impulsiveness, as an important component of personality, mediated the association between executive functions and the BPN of autonomy. In this study, the pre-season results showed that if a player shows better inhibition (Stroop time) and planning (TMT-A correct answers), lower impulsiveness (BIS-11 Total) may be predicted, which, in turn, is associated with better perception of self-control (PNT-A), and in post-season, when players showed a higher processing speed and cognitive flexibility, greater capacity of cognitive control for response planning (BIS-11 Total/no planning) may be predicted, but if athletes tend to think before acting (BIS-11 Motor impulsiveness), an association with better perception of self-regulation (Autonomy) is found. Our results suggest the importance of impulsiveness as a mediator between executive functioning and the psychological need for autonomy for optimal performance in athletes.

In both evaluation times, the levels of need thwarting of autonomy (PNT-A) allowed the prediction of the affective state, that is, the increase or decrease of depression symptoms, which may also be related to anxiety symptoms. The latter supports the previous findings that associate psychological need thwarting with symptoms of physical and psychological discomfort (Mars et al., 2017).

From a theoretical point of view, this study highlights the importance of assessing emotions and analyzing their role in sports success. Negative emotions are not always bad for cognitive functions in sports.

## Conclusion

In conclusion, our results showed the dynamics of sport-related psychological variables throughout the sport season in American football players. It is important to highlight the variations in cognitive functioning, impulsiveness, and BPN thwarting as a functional characteristic in the team at the end of the sports season, as well as the interaction between these variables for the improvement of sports performance. Our findings might be a useful tool for coaches to create selection profiles that support the prediction of sport success in the recruitment of sport talents. Further research is necessary to evaluate larger samples and other sport disciplines to increase the knowledge about the dynamics and association of sport-related psychological variables in sports performance.

## Data Availability Statement

All datasets generated for this study are included in the article/supplementary material.

## ETHICS STATEMENT

Ethical approval was not provided for this study on human participants because the study was presented to the team of coaches of the University, who in turn informed the authorities of the University. The patients/participants provided their written informed consent to participate in this study.

## Author Contributions

XO-J and YR-C contributed to the conception and design of the study. JS-L and YR-C organized the database and performed the statistical analysis. YR-C and JS-L wrote the first draft of the manuscript. YR-C, JS-L, XO-J, and JL-W wrote sections of the manuscript. All authors contributed to the manuscript revision, and read and approved the submitted version.

## Conflict of Interest

The authors declare that the research was conducted in the absence of any commercial or financial relationships that could be construed as a potential conflict of interest.
